# Thermal Conductive Polymer Composites: Recent Progress and Applications

**DOI:** 10.3390/molecules29153572

**Published:** 2024-07-29

**Authors:** Jianfeng Tan, Yuan Zhang

**Affiliations:** College of Intelligent Systems Science and Engineering, Hubei Minzu University, Enshi 445000, China

**Keywords:** thermal conductive, polymer nanocomposites, advanced application

## Abstract

As microelectronics technology advances towards miniaturization and higher integration, the imperative for developing high-performance thermal management materials has escalated. Thermal conductive polymer composites (TCPCs), which leverage the benefits of polymer matrices and the unique effects of nano-enhancers, are gaining focus as solutions to overheating due to their low density, ease of processing, and cost-effectiveness. However, these materials often face challenges such as thermal conductivities that are lower than expected, limiting their application in high-performance electronic devices. Despite these issues, TCPCs continue to demonstrate broad potential across various industrial sectors. This review comprehensively presents the progress in this field, detailing the mechanisms of thermal conductivity (TC) in these composites and discussing factors that influence thermal performance, such as the intrinsic properties of polymers, interfacial thermal resistance, and the thermal properties of fillers. Additionally, it categorizes and summarizes methods to enhance the TC of polymer composites. The review also highlights the applications of these materials in emerging areas such as flexible electronic devices, personal thermal management, and aerospace. Ultimately, by analyzing current challenges and opportunities, this review provides clear directions for future research and development.

## 1. Introduction

In the context of the ongoing trend towards miniaturization and high integration in microelectronics technology, improvements in device performance and lifespan are increasingly dependent on the effective implementation of advanced thermal management systems [1,2,3,4,5]. With the expansion of 5G technology and the prevalence of high-power devices, effective thermal conduction and dissipation have become key to ensuring stable device operation and extending their service lives, thus significantly highlighting the urgency of developing high-performance thermal management materials (TMMs) [6,7,8,9,10,11,12]. Moreover, thermal interface materials (TIMs), a critical subset of TMMs, are essential for managing heat in contemporary electronic devices by facilitating effective heat transfer and preserving electrical insulation [13,14,15]. The rapid development of technology has driven the demand for innovative high-performance materials, especially in high-temperature operating environments such as server rooms, electric vehicles, and aviation electronics, where higher demands are placed on TMMs [16]. Thermal management issues are not only crucial to the efficiency of device performance but also directly impact the efficiency of energy use and the sustainable development of the environment. For instance, in electric vehicles, the battery management system must effectively control and dissipate heat generated during the charging and discharging processes, failing which may decrease battery efficiency, shorten its lifespan, and even pose safety risks [17,18,19,20,21]. The development of new thermally conductive materials is thus not only a technical requirement for enhancing device performance and stability but also a response to the societal needs for environmental protection and efficient use of energy. Additionally, with the emergence of wearable devices and flexible electronics, there is an acute increase in demand for composite materials that possess good mechanical properties and can effectively manage thermal energy [22,23,24,25]. These devices often operate in environments sensitive to temperature changes, requiring materials that not only exhibit good thermal conductivity (TC) but also sufficient flexibility and stretchability to accommodate device bending and stretching.

Thermal conductive polymer composites (TCPCs) have secured an important position in modern thermal management systems due to their light weight, low cost, and excellent electrical insulation properties [26,27,28,29,30]. These materials combine the ease of processing of polymer matrices with the unique physicochemical properties of nanoscale enhancers, particularly excelling in enhancing TC and mechanical stability. However, these composites also face certain non-negligible limitations in practical applications, primarily their inherent TC often being lower than that of metallic materials, which limits their use in applications requiring very high TC [31,32,33]. Although the thermal performance of TCPCs can be enhanced by adding conductive fillers such as carbon nanotubes and graphene, this addition tends to increase the material’s interfacial thermal resistance and decrease its overall mechanical strength [34,35]. To overcome these challenges, researchers have developed various strategies, including optimizing the type, morphology, distribution, and surface treatment of fillers, to improve the thermal performance of polymer composites [36,37,38,39,40]. The implementation of these strategies has led to certain advancements; for instance, improving the compatibility between nanofillers and polymer matrices can significantly reduce interfacial thermal resistance, thereby enhancing the overall TC of the composites [41,42,43,44,45]. Additionally, modern techniques also allow for precise control over the dispersion and directional alignment of nanofillers, further enhancing the composite’s TC [46,47,48]. Despite ongoing challenges in enhancing thermal performance, the broad application prospects of TCPCs in sectors such as electronics, personal thermal management, and aerospace remain promising [49,50,51,52]. These materials not only meet the needs of modern electronic devices for efficient thermal management but are also considered key to enhancing the overall performance of devices due to their excellent electrical insulation and mechanical properties. Therefore, despite the challenges, the research and development of TCPCs continue to be a hot topic in the field of materials science.

This review article aims to comprehensively assess the latest advancements in TCPCs, particularly their potential applications in modern high-tech sectors. The article first details the thermal conduction mechanisms of TCPCs, exploring various factors that influence their thermal performance, including but not limited to the intrinsic properties of the polymers, interfacial thermal resistance, and the thermal properties of the fillers. Additionally, the article summarizes the main methods currently employed to enhance the TC of these materials and discusses specific application cases in emerging sectors such as personal thermal management and aerospace. While extensive research has been conducted on the preparation, performance enhancement, and mechanistic analysis of TCPCs, this paper additionally seeks to address the gaps related to their practical application details and requirements. Through an in-depth analysis of the current challenges and opportunities, this review seeks to provide clear directions and insights for future research and development of TCPCs. It is hoped that this systematic analysis will assist materials scientists and engineers in selecting and designing new thermal conductive materials, providing scientific foundations and innovative ideas, while also helping readers to understand the application potential and practical needs more fully in various fields.

## 2. Thermal Conductive Mechanism

### 2.1. Intrinsic Thermal Conductive Polymers

**Figure 1:**
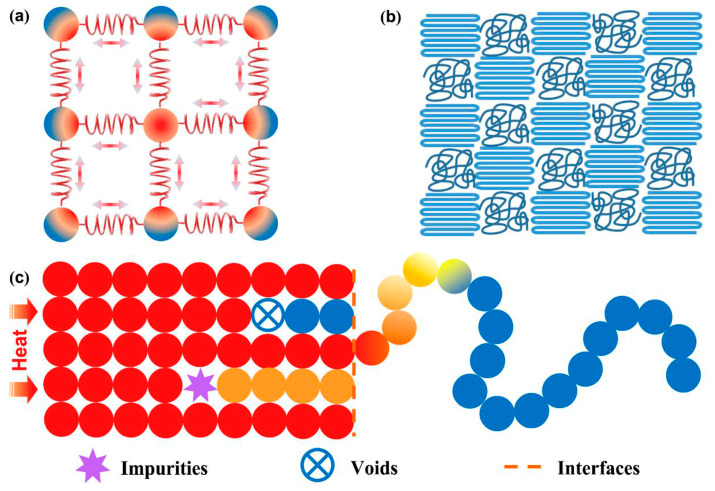
(**a**) Illustration of molecular chains or atoms vibration by mass and spring system; (**b**) crystalline and amorphous regions in polymers; (**c**) factors causing phonon scattering in polymers [66].

In the realm of solid materials, thermal conduction primarily operates via electrons and phonons, with the dominant mechanism depending on the material type. In metals, the high mobility of free electrons not only facilitates efficient electrical current transfer but also rapid heat dissipation. This phenomenon is explained by models such as the Drude model, where the movement and scattering of electrons determine the efficiency of thermal conduction [53,54,55]. Unlike metals, the thermal conduction mechanism in polymers predominantly relies on phonons, which are vibrations of the molecular structure (Figure 1) [56,57,58]. Due to the near absence of free electrons in polymers, heat transfer is significantly influenced by the tangled nature of molecular chains, the structure of long-chain molecules, and the presence of micro-defects. In amorphous polymers, phonon transport faces increased scattering due to the lack of long-range ordered structures, resulting in lower thermal conductivity. However, in crystalline polymers, while the ordered lattice structure can enhance phonon transport efficiency, the presence of irregular structures and defects within the molecular chains, such as chain folding, grain boundaries, and microvoids, continues to cause phonon scattering and affect thermal conduction [59,60,61].

Given the inherently low TC of polymers, material scientists have developed various methods to enhance their thermal properties, including the development of TCPCs and optimization of their molecular structures through design. These innovations not only enhance the thermal performance of polymers but also expand their applications in advanced manufacturing, energy transmission, and cooling of electronic devices [62,63,64,65]. Through a deeper understanding of the intrinsic thermal properties of polymers and technological innovation, the limitations in specific applications can be effectively addressed, thereby expanding their use in modern technology.

### 2.2. Filled Thermal Conductive Polymer Composites

#### 2.2.1. Thermal Conductive Path Theory

**Figure 2:**
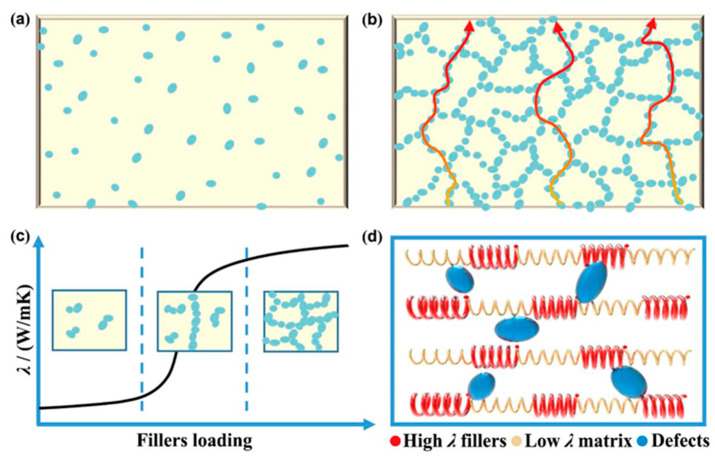
(**a**) “Sea-island” in low fillers loading; (**b**) thermal conduction paths in high fillers loading; (**c**) percolation phenomenon; (**d**) thermoelastic coefficient theory [66].

In TCPCs enhanced with fillers, thermal conduction pathways are established through the incorporation of thermal conductive fillers into the polymer matrix [66,67,68]. The distribution of these fillers and their interfacial characteristics with the matrix are critical factors affecting the thermal performance of the composites. At low filler concentrations, as shown in Figure 2a, the conductive particles remain dispersed, isolated by the matrix, which contributes to considerable interfacial thermal resistance and keeps the composite’s TC relatively low [69,70,71]. As the filler concentration increases, as depicted in Figure 2b, particles begin to make direct contact, gradually forming a continuous thermal network. the conductive particles remain dispersed, isolated by the matrix, which contributes to considerable interfacial thermal resistance and keeps the composite’s TC relatively low [71,72]. However, if the thermal network does not form along the direction of heat flow, high thermal resistance persists, limiting improvements in thermal conductivity.

Additionally, at lower filler content, the common “sea–island” structure isolates the fillers within the polymer matrix, creating multiple isolated conductive islands. In such structures, due to the high interfacial thermal resistance, phonon transmission primarily occurs through these interfaces, resulting in limited enhancement of TC [73,74,75]. As the filler content increases, direct contact between filler particles becomes more frequent, gradually forming effective thermal conduction paths that allow heat to flow along paths of lower thermal resistance, significantly enhancing the composite’s thermal conductivity. The development and refinement of these pathways are critical to advancing high-performance TCPCs. Consequently, optimizing filler distribution and minimizing interfacial thermal resistance are essential strategies for significantly boosting the thermal performance of polymer composites, addressing the growing needs for adept thermal management.

#### 2.2.2. Thermal Conductive Percolation Theory

Percolation theory, originally used to explain the electrical conductivity phenomena in conductive composites, has been extended to analyze the thermal conduction behavior in TCPCs. This theory posits that when the filler content reaches a certain critical threshold, the properties of the composite undergo significant changes due to the formation of a continuous network by the filler particles, causing abrupt changes in thermal conductivity, as shown in Figure 2c [76,77,78,79]. At lower filler contents, with fillers dispersed uniformly within the polymer matrix and not forming a network, the “sea–island” structure is formed, and TC increases only gradually with filler content. However, surpassing the percolation threshold causes the fillers to configure into a “sea–sea” structure, triggering a marked enhancement in thermal conductivity. Nonetheless, in thermal conductive composites, the percolation point may not always be clearly discernible, and the variation in TC (λ value) might not exhibit a sudden spike. This phenomenon can be attributed to substantial phonon scattering at the interfaces between the fillers and the polymer matrix, which introduces notable interfacial thermal resistance.

Although percolation theory is somewhat applicable in explaining the thermal behavior of filled TCPCs, it has limitations in realistically interpreting the λ value changes of these materials. Near the percolation transition point, the increase in TC tends to be more gradual compared to the often-dramatic rise in electrical conductivity observed in these materials. Notably, significant thermal percolation effects are typically evident only with the incorporation of high-TC fillers. In such scenarios, the thermal resistance between the fillers is markedly lower than the interfacial thermal resistance between the fillers and the polymer matrix. Consequently, the formation of a continuous network of these fillers substantially boosts thermal conductivity. However, the complexities of phonon scattering and interfacial thermal resistance mean that percolation theory’s explanatory power and predictive accuracy for practical applications remain limited. However, due to the complexities of phonon scattering and interfacial thermal resistance, the explanatory power and predictive accuracy of percolation theory in practical applications require further research and validation.

#### 2.2.3. Thermoelastic Coefficient Theory

Thermoelastic coefficient theory views filled TCPCs as a macroscopic whole, emphasizing that their TC is influenced by macroscopic properties such as elastic coefficients and modulus changes, as depicted in Figure 2d. This theory posits that by enhancing the thermoelastic coefficient in the material, i.e., optimizing phonon transmission efficiency, TC can be significantly improved. Specifically, by incorporating high-TC fillers like carbon nanotubes or graphene, an effective thermal conduction network is formed. This setup significantly reduces phonon scattering and interfacial thermal resistance, thereby substantially enhancing the composite’s thermal conductivity. However, increases in TC are usually not abrupt, as phonon transmission is still subject to the base properties and interfacial characteristics of the matrix. Consequently, enhancing the thermal performance of polymer composites hinges on optimizing the types, concentrations, and spatial distribution of fillers, alongside refining the interfacial interactions between the fillers and the matrix [80,81].

## 3. Key Factors Influencing the TC of Polymer Composite Materials

### 3.1. Key Factors Affecting the TC of Polymer Composites

#### 3.1.1. Intrinsic TC of the Polymer

**Figure 3:**
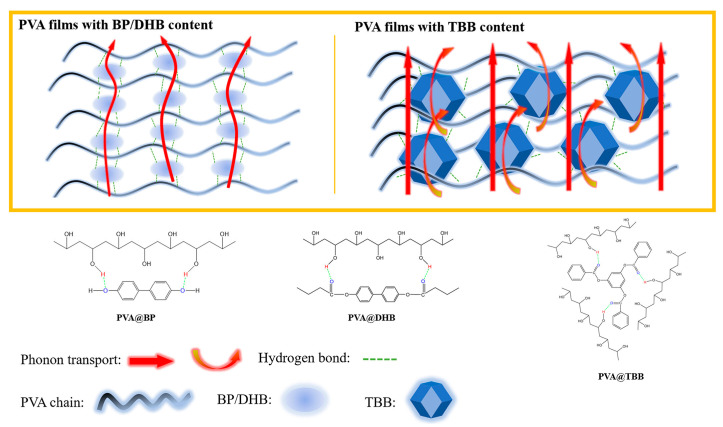
Microscopic ordered structures and the corresponding phonon transport model of polyvinyl alcohol (PVA) composite films [86].

Understanding the crystalline and amorphous states in polymers is crucial for grasping their TC [82,83,84]. In polymers, macromolecular segments typically consist of many molecular chains that form through entanglement and folding, containing both crystalline and amorphous regions. The crystalline regions, where molecular chains are uniformly oriented and tightly packed, exhibit clear periodicity. This ordered structure allows for efficient phonon transmission, thus enhancing the material’s thermal conductivity. Conversely, in the amorphous regions, the arrangement of molecular chains is more irregular, leading to structural defects and an increase in interfaces, which in turn increase phonon scattering and reduce the TC of the polymer bulk. The thermal performance of polymers is also significantly influenced by their molecular structure, including factors such as the degree of crystallinity, orientation, polarization of polar groups, number of atoms per unit volume, bond strength, molecular chain structure (including main chain bonds, side chains, and inter-chain coupling), and cross-linking density (Figure 3) [85,86]. For example, mechanical stretching can transform the amorphous structure of polymer chains into a crystalline structure with uniform orientation, which not only reduces phonon scattering but also effectively enhances TC through a straight, ordered polymer backbone and periodic structure of the molecules. In practical applications, research by Tu et al. employed a dual chemical–physical cross-linking and stretching orientation strategy to prepare highly thermally conductive, mechanically robust cellulose/hydroxylated boron nitride (BN) nanosheet composite films [87]. Furthermore, the number and polarization degree of polar groups in polymers directly impact TC [88]. The more polar groups present and the higher their degree of polarization, the higher the TC of the polymer.

In strategies to enhance the intrinsic TC of polymers, both the nanotemplating method and electrospinning have shown their unique effectiveness [89,90,91]. The nanotemplating method involves melting the polymer material and infiltrating it into a porous template, which orders the molecular chains as they flow into the nanoscale pores, thereby increasing the TC of the nanofibers. On the other hand, the electrospinning technique uses electrostatic forces to draw a polymer solution from the tip of an injection needle, forming polymer nanofibers where most molecular chains are aligned along the fiber axis. This orderly arrangement significantly enhances the TC along the fiber direction. By altering the structure of polymer molecules and chains, special physical structures can be obtained through synthesis and molding processes, thereby improving their thermal conductivity. Additionally, owing to their high aspect ratio, nanofibers provide a larger surface area, shorter thermal conduction paths, and increased interfacial heat transfer areas. These methods not only include the aforementioned high-stretching, electrospinning, and templating methods but also involve tuning the physical properties of polymers by optimizing molecular weight and the number of chains ends to achieve higher thermal conductivity. This series of research and development efforts continually pushes the application of polymer materials in the field of thermal conductivity, especially in the widespread use within electronic packaging domains.

#### 3.1.2. Filler Properties

In the development of polymer composite materials, the selection, morphology, and spatial distribution of thermal conductive fillers within the matrix are pivotal factors that dictate the thermal performance of the composites [92,93,94,95,96]. These fillers are classified into carbon-based, inorganic, metallic, and specially functionalized hybrid fillers, each contributing uniquely to the composites due to their distinct physical and chemical properties. Carbon-based fillers are frequently used in high-TC composites that require electromagnetic shielding due to their excellent thermal and electrical properties. Inorganic fillers like BN and aluminum oxide are suited for applications requiring high thermal stability and good insulation properties. Metallic fillers, including silver (Ag), copper (Cu), and aluminum (Al), are extensively used in efficient thermal management systems for their extremely high thermal conductivity, although they are prone to oxidation and have higher thermal expansion coefficients at elevated temperatures.

The morphology of fillers—including zero-dimensional nanoparticles, one-dimensional nanowires, two-dimensional layers, and three-dimensional networks—significantly impacts thermal conductivity. Zero-dimensional fillers typically struggle to form effective thermal conduction paths due to their point-like structure. In contrast, one-dimensional and two-dimensional fillers, with their longer thermal transfer paths and larger surface contact areas, are more effective at constructing thermal networks and reducing thermal contact resistance. Three-dimensional fillers can form continuous thermal conduction channels in multiple directions, greatly reducing thermal resistance. The size of the fillers is also critically important. Although smaller-sized fillers can increase the interface area and phonon scattering sites, overly small sizes may lead to uneven distribution and aggregation within the polymer matrix, creating thermal bridging effects that impact overall thermal conductivity. Larger-sized fillers aid in forming continuous thermal conduction paths, but their distribution uniformity and interface quality within the polymer are equally crucial. More importantly, the thermal expansion coefficients and the impact on the conductivity due to the expansions in different fillers vary on the basis of the dimensionality.

Furthermore, the orientation of fillers significantly influences the TC of the composites. Fillers oriented by electric fields, magnetic fields, or mechanical forces can form efficient thermal conduction channels in specific directions. For example, Li et al. achieved high TC in epoxy composites by orienting carbon fibers under an applied stress field and curing them in situ in an epoxy resin. With a carbon fiber content of 46%, these composites achieved a thermal conductivity 171 times higher than that of pure epoxy resin [95]. Similarly, Shang et al. used an alternating vacuum filtration method to produce cellulose nanofiber/BN nanosheet/Ti_3_C_2_T_x_ MXene films. With a 70 wt% BN nanosheet content, the films exhibited an in-plane TC of 19.97 W·m^−1^·K^−1^, underlining their potential for use in highly integrated electronic devices [96]. In the fields of microelectronics and high-performance materials, comprehensive consideration of the choice, morphology, size, surface treatment, and orientation of fillers is essential for designing and manufacturing polymer composites.

#### 3.1.3. Interfacial Thermal Resistance

Interfacial thermal resistance significantly impedes the transfer of thermal energy. In polymer composites, the transfer of heat across interfaces encounters obstacles, often causing a sharp drop in temperature at these interfaces. This phenomenon is primarily due to the vibrational, acoustic, and modulus mismatch during phonon transmission, which causes severe scattering of phonons, significantly reducing their mean free path and thus decreasing the λ of the composites. Poor thermal contact between the filler and the matrix is a core reason for high interfacial thermal resistance; therefore, improving the thermal transfer performance of these microscopic interfaces is a key strategy [43].

### 3.2. Strategies to Enhance the TC of Polymer Composite Materials

In enhancing the thermal performance of composite materials, scientists have primarily adopted two strategies [97,98,99,100,101,102,103,104,105,106,107,108]. Firstly, researchers have functionalized the interfaces by adding functional groups to the filler surfaces through chemical or physical means. Secondly, by precisely controlling the microscopic structural parameters such as the size, shape, dispersity, and orientation of the fillers, more optimized thermal networks can be constructed. Research on strategies to enhance the TC of polymer composite materials is summarized in Table 1. These strategies not only enhance the functionality of the composites but also provide key technical support for designing efficient thermal management systems.

#### 3.2.1. Interfacial Functionalization Reduces Interfacial Thermal Resistance

**Figure 4:**
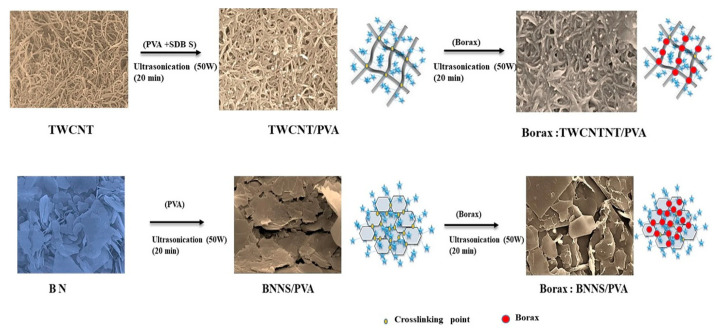
Enhancement mechanism through reinforced crosslinking and formation of thermal transport network by adding borax [100].

Interface functionalization involves the introduction of functional groups or layers onto the filler surface through chemical or physical methods, improving the compatibility of and interfacial bonding strength between the filler and the matrix [98,99,100,101,102]. This approach effectively reduces phonon scattering at the interface, enhancing phonon transmission and thereby lowering interfacial thermal resistance. From a microscopic mechanism perspective, interface functionalization typically includes covalent and non-covalent functionalization. Covalent functionalization involves forming stable chemical bonds on the filler surface, which also helps improve the dispersity of fillers and reduce agglomeration. However, it is important to note that excessive chemical modification may damage the surface structure of the fillers. Non-covalent functionalization utilizes physical adsorption or weaker chemical bonds, such as hydrogen bonds, to maintain the structural integrity of the fillers. Chen et al. employed one-dimensional thin-walled carbon nanotube (1D-TWCNT) and two-dimensional boron nitride nanosheet (2D-BNNS) fillers to enhance the thermal properties of PVA (Figure 4). A critical aspect in boosting PVA’s thermal properties is the interfacial configuration strategy, which facilitates phonon transport and minimizes the intrinsic thermal property loss of the filler nanomaterials. The inclusion of borax enhanced the TC of the 1D-TWCNT/PVA nanocomposites by up to 14.5% with 4 wt.% borax and the 2D-BNNS/PVA nanocomposites by up to 30.6% with 2 wt.% borax [100].

Chen et al. innovatively developed a composite material by incorporating crushed carbon fibers into natural rubber. The surface of the carbon fibers was first modified with dopamine, which self-polymerized to provide an initial coating. Subsequently, a silane coupling agent (SCA), specifically KH590, was grafted onto this platform to enhance interfacial bonding between the filler and the matrix. Testing revealed that the modifications with KH590 resulted in TC and tensile strength increases of 71.3% and 73.3%, respectively, when compared to the unmodified rubber, thus marking substantial enhancements in material performance [101]. Bashir et al. advanced composite materials by hydroxylating hexagonal BN (h-BN) and carbon nanotubes (CNTs), subsequently creating a polyurethane crosslink between the interfaces of the fillers and the thermoplastic polyurethane (TPU) matrix. The synergistic effect of the mixed fillers, comprising only 60 wt% h-BN and 2 wt% CNT, yielded a remarkable TC of 4.52 W·m^−1^·K^−1^ [102]. In summary, interface functionalization techniques, by optimizing the microstructure and interfacial performance within the composites, significantly reduce interfacial thermal resistance, greatly enhancing the TC of the composites. These techniques not only provide new avenues for the development of high-performance composite materials but also hold significant practical application value in demanding fields such as electronic devices and energy systems. As materials science continues to advance, these techniques are expected to demonstrate their unique advantages in a broader range of applications in the future.

#### 3.2.2. Microstructural Design Governs the Formation of Thermal Conductive Pathways

**Figure 5:**
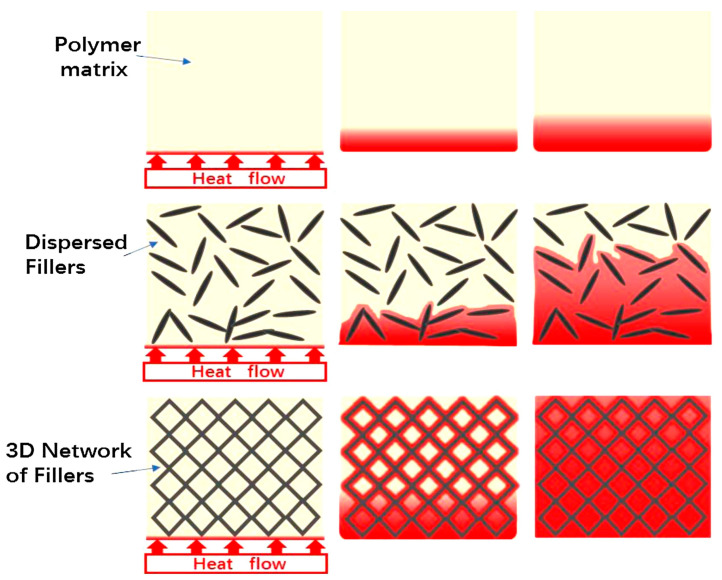
Schematic diagrams of heat transport in a pure polymer, a polymer composite filled with a traditional dispersed filler, and a composite filled with a 3D interconnected network of fillers [106].

Scientists have developed various microstructural control strategies to construct more optimized thermal networks [103,104,105,106,107,108,109,110]. The size and morphology of fillers are crucial for forming effective thermal pathways. Smaller-sized nanofillers can form denser networks within the polymer matrix, thus enhancing thermal conductivity. For instance, carbon nanotubes and graphene, with their unique one-dimensional and two-dimensional structures, can form continuous thermal networks across the composite material (Figure 5). Additionally, fillers of different morphologies, such as spherical, flake-like, or fibrous, also affect their orientation and dispersity within the matrix, further influencing the thermal conductivity.

**Figure 6:**
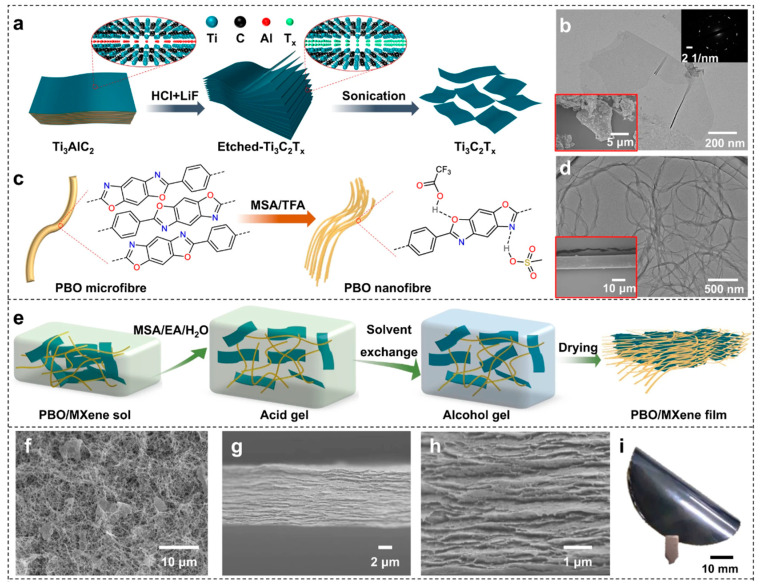
(**a**) Illustration for Ti_3_C_2_T_x_ MXene prepared from Ti_3_AlC_2_ MAX precursor by HCl/LiF etching. (**b**) TEM image of MXene nanosheets (insets: electron diffraction pattern of MXene and SEM image of Ti_3_AlC_2_ MAX). (**c**) Illustration for PBO nanofibres prepared from commercial PBO fibres by MSA/TFA exfoliation. (**d**) TEM image of PBO nanofibres (inset: SEM image of PBO fibre). (**e**) Illustration for the nacre-inspired PBO/MXene films prepared by the sol-gel-film conversion approach including a proton-consumption homogeneous gelation process. (**f**–**h**) SEM images of (**f**) the freeze-dried PBO/MXene gel and (**g**,**h**) the cross-section of PBO/MXene film. (**i**) The optical photograph of PBO/MXene film [107].

Uniform dispersion of fillers is another key factor in achieving high thermal conductivity. Unevenly dispersed fillers can cause interruptions in the thermal conduction path, thereby reducing thermal efficiency. Techniques such as high shear mixing and ultrasonic dispersion can effectively improve the uniform distribution of fillers in the polymer matrix. Moreover, the orientation of fillers significantly impacts TC performance. For example, aligning long fibers or flake-like fillers in a specific direction can create more effective thermal conduction paths. Additionally, mixed filler strategies employ various types, shapes, and sizes of thermal conductive fillers, orchestrated through tailored microstructural designs to establish effective thermal conduction pathways. Research shows that mixed filler systems can achieve high TC at lower total filler loadings, significantly outperforming traditional single-filler systems. At the microscale, by precisely controlling the size, morphology, dispersity, and orientation of fillers, a continuous and effective thermal network can be constructed. Liu et al. have synthesized a high-performance poly(p-phenylene-2,6-benzobisoxazole) (PBO)/MXene nanocomposite film using a sol–gel film conversion method (Figure 6). The resultant film benefits from an optimized brick and mortar structure, enhanced by the strong bridging and caging effects of the fine PBO nanofiber network on the MXene nanosheets. Critically, the synergistic orientation of the PBO nanofibers and MXene nanosheets imparts an in-plane TC of 42.2 W·m^−1^·K^−1^ to the film [107]. This requires meticulous design and optimization of the material’s microstructure, such as predicting the impact of internal structures on TC through computer simulations and utilizing advanced microscopy techniques for precise measurement and analysis of material structures [108].

In practical applications, the processing methods for TCPCs are also crucial. Common processing methods include templating, melt extrusion, injection molding, and hot pressing [109,110,111]. These methods need to consider the thermal stability and rheological properties of the materials and ensure uniform dispersion and appropriate orientation of fillers during processing to maintain the TC performance of the materials.

## 4. Applications of Thermal Conductive Polymer Composites

### 4.1. Electronic Devices (Wearable Electronics)

With technological advancements, the integration and power density of electronic devices, such as chips, are continually increasing, leading to serious issues with heat accumulation. TCPCs have become indispensable materials in modern electronic devices and systems due to their excellent performance in thermal management. TCPCs combine the high TC and electrical insulation properties of inorganic materials with the lightweight, easy processing, and low-cost advantages of organic polymers, making them widely used in underfill materials and TIMs [112,113,114,115,116,117,118,119,120]. Epoxy resin composites, due to their low cost and ease of processing, hold a significant position in electronic packaging. Xiao et al. proposed a simple, environmentally friendly method to prepare high-TC epoxy resin/BN nanosheet composites. Due to the interconnected network, this composite material exhibits excellent thermal performance in all directions, promising substantial applications in thermal management of electronic devices. Furthermore, the thermal stability of the composite material was significantly enhanced, with the T 20 increasing by 227.3 °C compared to pure epoxy resin [119]. Yan et al. prepared high-TC epoxy resin composites using 60 wt% of f-Al_2_O_3_, 3 wt% of MWCNTs, and 8 wt% of SiO_2_ NPs. Through uniform dispersion and a synergistic mixing effect, various components were combined to construct an interconnected thermal network, achieving efficient thermal conduction. The composite material exhibited an out-of-plane TC of 1.73 W·m^−1^·K^−1^, suitable for thermal management components in electronic devices [120]. Liu et al., based on synthesized epoxy vitrimer and BN (BN) nanosheets, fabricated a fully recyclable high-TC thermal TIM. Simple hot pressing, with 40 wt% BN filling, increased the TC to 3.85 W·m^−1^·K^−1^, thirty times that of the original epoxy resin, significantly enhancing the heat dissipation of electronic devices. Epoxy/BN-40 wt% exhibited a high-onset thermal degradation temperature of approximately 280 °C, indicating excellent thermal stability [121].

**Figure 7:**
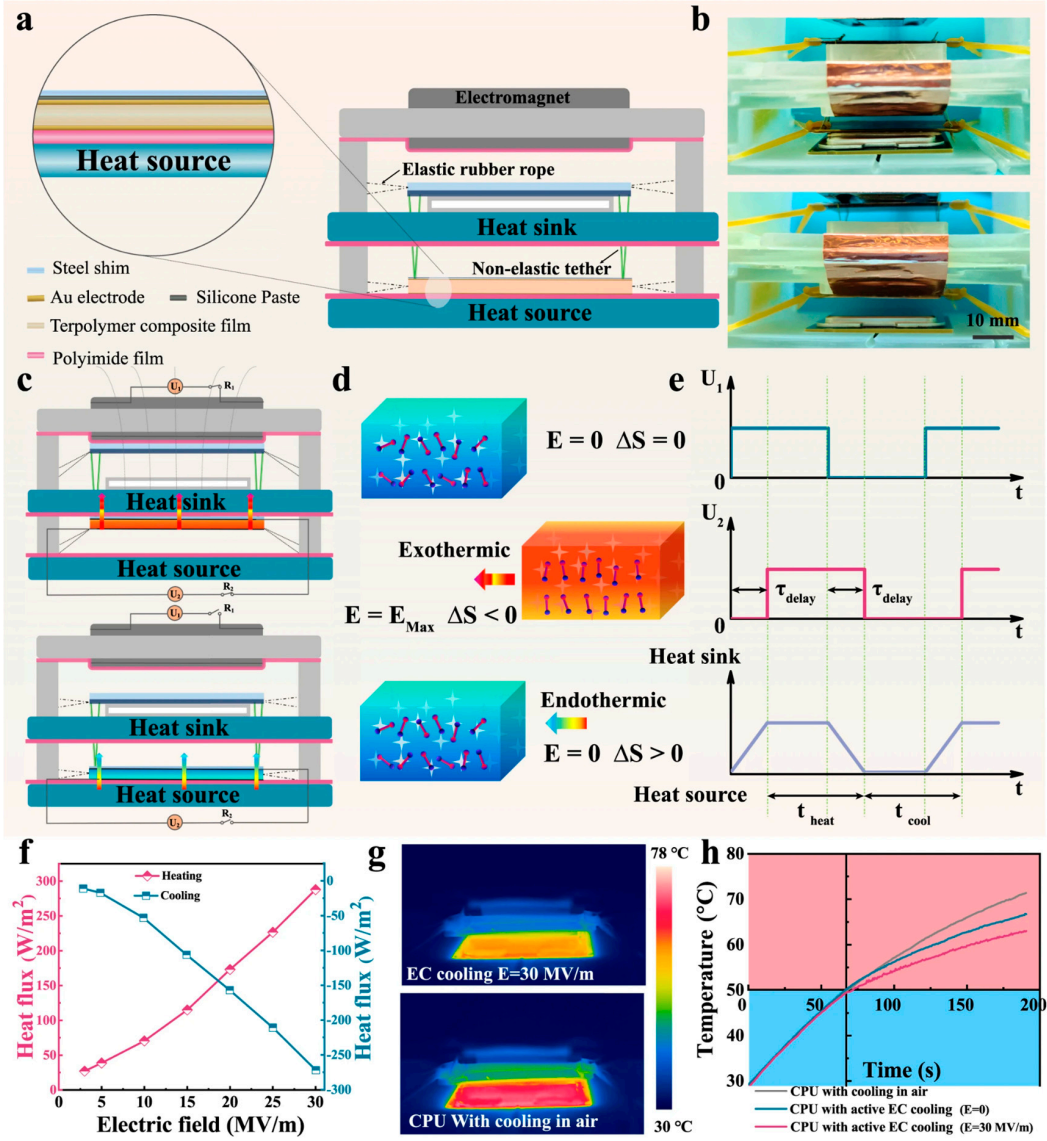
(**a**) Schematic illustration of the electrocaloric polymer stack and solid-state cooling device. (**b**) Photograph of the active EC device; the elaborated main framework was obtained by 3-D printing. (**c**) The schematic shows how an electromagnetic field drives an electrocaloric polymer stack to move heat from a heat source to a heat sink. The high-speed heat transfers from heat source to sink can be achieved by associating the active cooling of electrocaloric polymer stack with the heat dissipation cycle. (**d**) The working mechanism of the ECE based on the change in dipole entropy. (**e**) Time domain illustration of the cooling cycle. (**f**) The maximum heat flux of the electrocaloric stack on the heating and cooling side versus the applied electric field is measured by a heat flux sensor at an operation frequency of 0.1 Hz. (**g**) Infrared thermal images of CPU in active electrocaloric cooling. (**h**) Temperature versus time curves of CPU in air, by active electrocaloric cooling (U1 = 12 V at 1 Hz, U2/d2 = E = 0 MV m^−1^; d2 is the thickness of the electrocaloric cooling stack) and active electrocaloric cooling (U1 = 12 V at 1 Hz, E = 30 MV m^−1^) [123].

Yan et al. proposed an efficient method for preparing ultra-high-aspect-ratio hexagonal BN nanosheets (BNNSs) based on microfluidic technology. Achieving a length-to-width ratio of approximately 1500 with minimal defects, BNNS composite films prepared with PVA exhibited an in-plane TC of 67.6 W·m^−1^·K^−1^ at 83 wt% BNNS content, a TC enhancement of about 35,500 times. Used as a heat sink for high-power LED modules, this composite film showed excellent heat dissipation efficiency [122]. Li et al. present an innovative interpenetrating architecture that integrates an electrocaloric polymer with highly thermally conductive pathways (Figure 7). This configuration achieves a 240% increase in electrocaloric performance and a 300% enhancement in the polymer’s thermal conductivity. Utilizing this electrocaloric composite, we fabricated a scaled-up prototype device specifically designed for single heat spot cooling of a 5G chip, incorporating electromagnetic actuation [123].

Due to their compact designs, wearable devices are prone to heat accumulation under high-frequency operating conditions. Since these devices come into direct contact with human skin, their temperature should not significantly exceed that of the human body, necessitating the use of high-TC materials for packaging to ensure device cooling and safety [124,125,126]. Miniaturized, high-power-density wearable electronics demand advanced thermal management, excellent flexibility, and superior permeability. Balancing these requirements is challenging due to the inherent conflict between thermal conductivity and the properties of flexibility and permeability. Despite these challenges, significant progress has been made in managing the thermal properties of flexible wearable devices through the use of thermal conductive polymer nanocomposites. High-TC elastomer nanocomposites not only ensure rapid dissipation of heat generated within the device but also quickly respond to artificial thermal feedback, providing heating and cooling functions.

**Figure 8:**
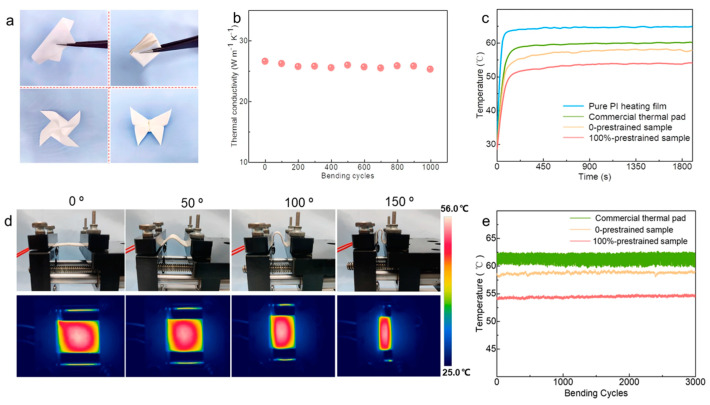
(**a**) The flexibility of the composite. (**b**) In-plane thermal conductivity of the 100%-prestrained composite in response to 1000 bending cycles. (**c**) Surface temperature variations of the flexible PI heating film against time. (**d**) Temperature fluctuation of the flexible PI heating film with the 100%-prestrained composite under a bending angle of 0−150°. (**e**) Temperature fluctuation of the flexible PI heating film with different TIMs under more than 3000 bending cycles [131].

For example, Ma et al. employed emulsion electrospinning to create smart temperature-controlled nanofibers, incorporating lauric acid PCM, carbon nanotubes, and zinc oxide to endow them with excellent TC (0.665 W·m^−1^·K^−1^) and UV resistance. The polyvinyl alcohol base ensures flexibility, with a break elongation of 25%. A non-polluting polydimethylsiloxane coating provides hydrophobicity and self-cleaning properties, making it suitable for smart garments and protective fabrics [127]. Chen et al. reported a method for creating flexible, breathable composite materials by applying high-TCBN nanosheets BNNS onto a patterned electrospun TPU fiber mat grid. This composite material significantly enhanced TC while maintaining breathability. When integrated into flexible devices, its saturation operating temperature was significantly reduced, with surface temperature fluctuations less than 0.5 °C after more than 2000 bending–release cycles [128]. Tan et al. developed a stretchable strain sensor with exceptional thermal management performance. Its unique feature is a cast TPU-BN nanosheet layer, which not only enhances TC but also rapidly transfers heat to the environment, while the porous electrospun TPU film acts as a thermal insulator, reducing the real-time saturation temperature by 32%. [129]. Wu et al. developed high-TC regenerated cellulose/BNNS composite yarns through wet spinning and confinement-induced self-assembly, achieving a TC of 9.22 W·m^−1^·K^−1^, 4.5 times that of traditional materials. This composite yarn was woven into textiles and integrated with a thermoelectric generator (TEG) to effectively harvest body heat, continuously powering wearable devices [130]. Guo et al. introduced highly flexible composites consisting of aligned and overlapping interconnected boron nitride nanosheets (BNNSs) assembled in wrinkle structures (Figure 8). These composites exhibited a high in-plane thermal conductivity exceeding 26.58 W·m^−1^·K^−1^ and enhanced through-plane conduction, improving with increased pre-strain. During an extensive bending test exceeding 3000 cycles, the maximum temperature fluctuation in the flexible device using a 100%-prestrained composite remained within 0.9 °C, which is less than one-third of that observed with a commercial thermal pad [131].

Polyimide (PI) materials are recognized for their exceptional heat resistance and stability, making them highly suitable for applications in the electronics industry. Employing in situ polymerization, wet spinning, and subsequent heat drawing techniques, Fang et al. achieved uniform dispersion of nanofillers and covalent bonding between polydopamine (PDA)@BNNS and the PI matrix, with high orientation of the fillers. This process resulted in notable enhancements in thermal diffusion, while a 10 wt% nanofiller addition achieved a TC of 3.44 W·m^−1^·K^−1^. The filament containing 0.5 wt% PDA@BNNS exhibits a maximum tensile strength of 2.9 GPa. The good dispersion of the filler and the strong interfacial interactions between PDA@BNNS and the PI matrix, induced by chemical bonds, are expected to play a significant role in enhancing the mechanical properties and thermal conductivity of the composite fibers [132]. In another study, Yoon et al. focused on improving the TC of PI by enhancing chain orientation and intermolecular interactions. Their research demonstrated that the thermal conductivities of a single electrospun PI nanofiber (INF-PI) and a hot-pressed nanofiber PI film (HP-PI) were 0.44 and 0.98 W·m^−1^·K^−1^ at room temperature, respectively. The electrospinning process was found to improve chain orientation, while the high pressure and heat treatment during hot pressing facilitated the development of π–π interactions, significantly increasing the TC of the HP-PI. Even after 3000 cycles, no cracks or delamination between the alternately stacked layers were observed in the film, demonstrating the exceptional flexibility and durability of the synthesized material [133]. Additionally, Ruan et al. developed a novel high-TC liquid crystal PI (LC-PI) film by optimizing the liquid crystal range to align with the curing temperature. The in-plane and through-plane thermal conductivities of the LC-PI IV film at room temperature were 2.11 W·m^−1^·K^−1^ and 0.32 W·m^−1^·K^−1^, respectively, which are significantly higher than those of films cured outside the liquid crystal range. Simultaneously, the LC-PI film exhibited excellent mechanical and thermal properties, indicating its potential for application in high-temperature flexible electronics [134].

Room temperature fast-self-healing structures and functions of composite materials have broad application prospects; however, given the complexity of composite material structures and compositions, their self-healing faces challenges. Yu et al. developed a high-TC self-healing composite material, 2-[[(butylamino) carbonyl] oxy] ethyl ester (PBA)-polydimethylsiloxane (PDMS)/folded graphene film (FGf), capable of fully self-healing within 10 min at room temperature. This material exhibited high fracture tensile strength (2.23 ± 0.15 MPa) and TC (13 ± 0.2 W·m^−1^·K^−1^), with self-healing efficiencies reaching 100% and 98.65%, achieved based on supramolecular interactions between polymers and graphene [135]. Through this research and its applications, TCPCs are demonstrating significant value in the field of flexible electronic devices, with the potential for widespread adoption in many practical applications in the future. Whether in electronic packaging or wearable devices, the application prospects of TCPCs are very broad and are expected to have a profound impact on the development of electronic technology [136].

### 4.2. Energy Storage Devices

**Figure 9:**
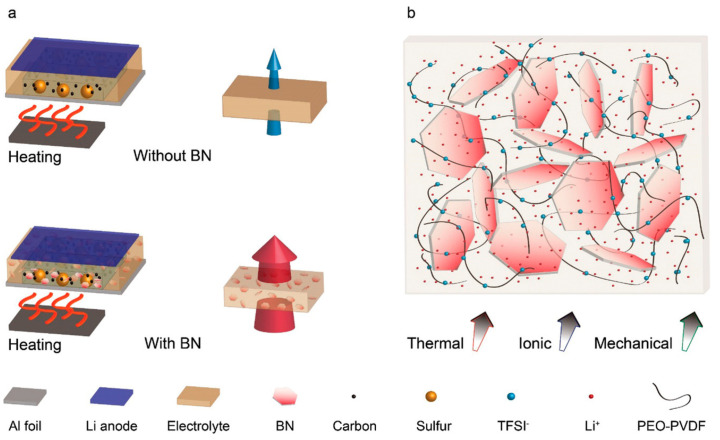
Rational design of solid-state polymer electrolyte with BN additive. (**a**) Illustration of solid-state Li-S batteries with polymer-based electrolytes and schematic comparison of heat transport through electrolytes with and without BN additives and (**b**) sketch of a composite electrolyte consisting of 2D BN flakes and a blended PEO-PVDF polymer with LiTFSI [140].

With the rapid advancement of electric vehicles and renewable energy, the demand for energy storage devices has surged, making lithium-ion batteries and supercapacitors focal points of attention. These storage devices generate substantial heat during charging and discharging cycles, and ineffective heat management can lead to performance degradation or even safety hazards. TCPCs, known for their excellent thermal conductivity, have become key materials in the thermal management of energy storage devices [137,138]. During charging and discharging, if the heat generated by batteries is not efficiently dissipated, it can lead to excessive temperatures that adversely affect their lifespan and performance. TCPCs are widely used in the TIMs and separators of batteries, aiding rapid heat dissipation and maintaining battery operation within safe temperature limits. For instance, Huang et al. developed a novel flexible composite material, styrene butadiene styrene@paraffin/expanded graphite (SBS@PA/EG). In battery application tests, the battery modules using this material maintained a maximum temperature below 46 °C during 5C discharges, with a temperature difference controlled within 4 °C, proving its efficiency and practicality in battery thermal management [139]. Yin’s research team developed an electrolyte with an improved thermal response by adding two-dimensional BN nanosheets to a polyethylene oxide-based electrolyte (Figure 9). The addition of BN not only enhanced the thermal stability of the electrolyte but also improved its ionic conduction and mechanical properties. This discovery provides a vital pathway for improving the safety and efficiency of solid-state batteries [140].

Guo and his research team successfully developed graphene-filled TPU composites during the 3D printing process, achieving an asymmetric alignment of graphene. This vertically aligned 3D printed structure exhibited an in-plane TC of 12 W·m^−1^·K^−1^ at a graphene content of 45 wt%, eight times higher than that of horizontally printed structures and significantly surpassing traditional particle-reinforced polymer composites. This substantial increase in thermal conductivity was primarily due to the preferred orientation of graphene and finely controlled print parameters of multi-scale dense structures [141]. Cheng et al. developed a ternary composite PCM battery thermal management structure based on expanded graphite/polymer/paraffin, featuring high TC and electrical conductivity. This structure utilizes a dual-layer design to preheat batteries using Joule heat, rapidly increasing the temperature (20.5 °C/min) at low temperatures and improving discharge efficiency by 35.5%. Additionally, at high temperatures, it acts as a cooling medium, keeping the battery temperature at 42.2 °C, optimizing battery performance and reliability [142]. Pei et al. developed ultra-light, double-sided BNNS/poly(acrylic acid) composite-modified separators employing a layer-by-layer (LBL) self-assembly method to enhance the TC of these composite separators. These separators demonstrated excellent PS shuttling and lithium dendrite suppression capabilities under high temperatures and temperature gradients, significantly enhancing the cycle stability and rate performance of Li-S batteries [143].

In supercapacitors, which are valued for their high power density, rapid charge–discharge rates, and long cycle life, TCPCs play a crucial role. These materials significantly enhance the electrochemical performance and electrical conductivity of supercapacitor electrodes [144,145]. Li et al. developed a cross-linked polymer nanocomposite incorporating BN nanosheets, exhibiting exceptional dielectric properties across a broad range of temperatures and frequencies. The inclusion of BN nanosheets notably boosted the TC of the material, enhancing its operational stability at elevated temperatures. Moreover, this nanocomposite is lightweight, photolithographically patternable, and exhibits excellent mechanical flexibility [146]. Chen’s research on ladderphane copolymers, materials with inherently low electrical conductivity under high electric fields and temperatures, demonstrated a unique self-assembling mechanism through π–π stacking interactions that form highly ordered arrays, resulting in an intrinsic vertical TC of 1.96 ± 0.06 W·m^−1^·K^−1^. These copolymer films effectively dissipate heat, thus exhibiting superb cyclic stability under challenging conditions [147]. TCPCs are revolutionizing energy storage device applications by effectively managing heat, which enhances device performance and safety and drives innovation in energy storage technology. As advancements continue, TCPCs are expected to further propel the development of energy storage technologies [148,149,150,151,152].

### 4.3. Transmission Systems

In the realm of power transmission systems, the thermal management capabilities of insulation materials are crucial for enhancing overall performance and extending the lifespan of equipment. Traditional low-conductivity insulation materials often lead to heat accumulation during operation, increasing the risk of failures and reducing efficiency. Recent advances in high-TC polymer composites have shown great potential to address these issues [153,154,155,156]. Improving the TC of cable insulation can effectively reduce internal cable temperatures, thereby enhancing the cable’s current carrying capacity and operational stability. This improvement not only reduces maintenance costs but also extends the cable’s lifespan. To address dielectric material distortions in cable accessories and thermal management issues, Chi et al. grew silver nanoparticles (Ag NPs) on hexagonal BN and then incorporated them into ethylene propylene diene monomer (EPDM) to form a Ag NPs/BN/EPDM composite material. Their studies showed that increasing the Ag NPs content enhanced both the nonlinear electrical conductivity and TC of the composite [157].

**Figure 10:**
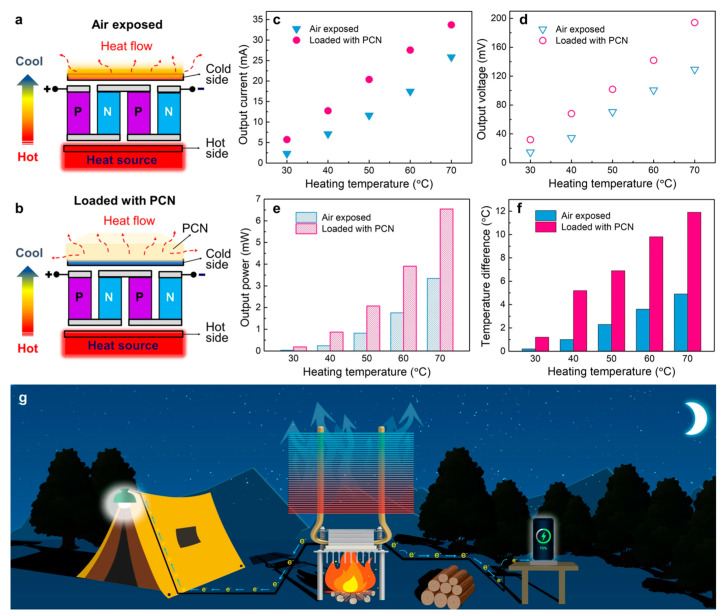
Thermal management application of PEG@TPU/BNNS-es film (with 32 wt% BNNSs content) in thermoelectric generator (TEG). (**a**) Schematic mechanism of TEG exposed to air (top) and (**b**) loaded with PEG@TPU/BNNS-es (PCN) (bottom). (**c**−**e**) Output current, output voltage and output power of TEGs with/without PCC at different heating temperature, respectively. (**f**) Temperature difference between the hot and cold sides versus heating temperature of TEGs. (**g**) Conception diagram of potential application of TEGs integrated with the PEG@TPU/BNNS-es film for outdoor activities [158].

Enhancing the TC of the main insulation materials can significantly lower the operating temperatures inside generators, thereby improving their output capacity and efficiency. Lin and others developed a phase change nanocomposite (PCN) using coaxial electrospinning, electrostatic spraying, and hot-pressing techniques (Figure 10). This advanced PCN film, containing 32 wt% of aligned and interconnected BN nanosheets, exhibited an extraordinary TC of 28.3 W·m^−1^·K^−1^. The successful implementation of this core-sheath strategy has positioned PCNs as ideal cooling solutions for high-power-density devices like 5G base station chips and thermoelectric generators, demonstrating significant potential in thermal management applications [158].

Transformers and other power devices, such as solid insulation switchgear and saturable reactors, also face overheating issues [159,160]. Wei and his team developed a thermofluidic coupled cooling model for saturable reactors to address thermal losses in high-voltage direct current systems. Their research indicated that increasing the TC of the epoxy resin insulation layer (from 0.8 to 1.2 W·m^−1^·K^−1^) significantly enhanced the cooling effect. This improvement effectively reduced core temperatures, validating the strategy of enhancing thermal dissipation efficiency by optimizing TC [161]. Overall, the development and application of high-TC polymer composites have brought revolutionary improvements to power transmission systems. These materials not only effectively resolve thermal management challenges but also propel the technology of power devices towards greater efficiency and reliability. As materials science continues to advance, these new materials are expected to play an increasingly critical role in future power systems.

### 4.4. Solar Energy

Solar thermal storage is crucial for enhancing the efficiency of solar energy utilization. PCMs are ideal for solar thermal storage due to their ability to absorb or release substantial amounts of heat during phase transitions. However, the low TC of PCM limits their application under high-temperature concentrated solar beams. Incorporating high-TC nano-fillers into PCM can significantly improve their thermal performance, thereby enhancing the efficiency of solar thermal storage systems [162,163,164]. In solar collectors and storage systems, PCMs are widely used for thermal energy storage and release. These materials provide latent heat by changing the state of the material without altering the temperature, effectively addressing the discontinuity of solar supply. However, the low TC of PCMs (about 0.2 W·m^−1^·K^−1^) can lead to hotspots under concentrated solar radiation, preventing uniform heat conduction throughout the receiver and leading to suboptimal energy conversion efficiencies and potential material structural damage due to overheating. Therefore, improving the TC of PCMs is key to optimizing solar thermal storage technology.

**Figure 11:**
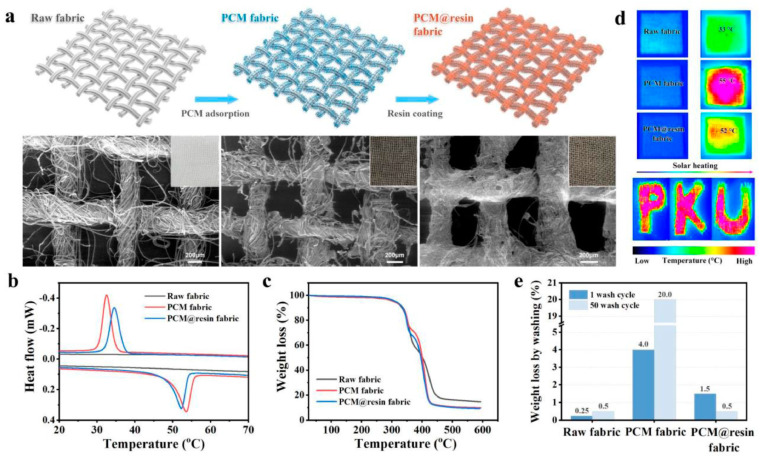
Solar heat storage fabric. (**a**) Schematic illustration of fabric treatment and corresponding SEM images. The inset of SEM images shows real photographs of corresponding fabric. (**b**) DSC thermograms of raw fabric, PCM fabrics and PCM@resin fabric. (**c**) TGA thermograms of raw fabric, PCM fabrics and PCM@resin fabric. (**d**) IR images of raw fabric, PCM fabrics and PCM@resin fabric before and after 2 min of solar irradiation at 80 mW/cm^2^. The PKU letter pattern is IR images of developed fabrics after 2 min of solar irradiation. (**e**) Bar graph showing fabric weight loss after washing [167].

To enhance the thermal performance of PCMs, researchers have experimented with incorporating various high-TC nano-fillers. Peng et al. developed an anisotropic graphene aerogel phase change composite with an optimized structure through directional freezing and high-temperature processing, achieving high-transverse (2.68 W·m^−1^·K^−1^) and longitudinal (8.87 W·m^−1^·K^−1^) thermal conductivities and a latent heat retention rate of 98.7% [165]. Mo’s research team developed a novel polyethylene glycol (PEG 2000)-based PCM composite using a Ti_3_C_2_T_x_@PVA foam skeleton as a carrier. By vacuum impregnating and freeze-drying techniques, PEG was introduced into the 3D interconnected skeleton, significantly increasing the TC to 0.428 W·m^−1^·K^−1^, which is 4.2 times that of pure PEG 2000. This structure not only enhanced TC but also exhibited excellent thermal and chemical reliability [166]. Aftab et al. developed a highly effective solar thermal storage solution by embedding phosphorene nanoflakes (PNFs) derived from high-quality bulk black phosphorus (BP) single crystals into a polyurethane (PU)-based solid–solid PCM matrix using a precipitation/crystallization-induced encapsulation strategy (Figure 11). This method ensured uniform distribution of PNFs and enhanced the composite’s structural stability and thermal performance, providing an effective technological path for efficient solar thermal storage [167].

Gao et al. developed a 3D thermal conductive framework combining aramid nanofibers (ANF) and graphene nanoplates (GNP) through ball milling techniques to enhance interfacial interactions and utilized unidirectional freeze-casting to produce a highly oriented honeycomb porous structure. With a GNP volume fraction of only 4.26%, the PCM composite material achieved a TC of 3.9 W·m^−1^·K^−1^, greatly enhancing the rate and stability of thermal charging and discharging [168]. Moreover, the application of TCPCs in photovoltaic modules has also garnered wide attention. Photovoltaic cells, while converting solar energy, lose a significant portion of solar radiation as heat, reducing the efficiency of the cells. Incorporating high TC and electrically insulating fillers in the encapsulating materials can effectively enhance the thermal dissipation of solar cells [169]. Huang et al. utilized graphene nanoplates (GNs) as fillers and successfully fabricated a polyvinyl butyral (PVB) composite material with significantly enhanced TC through a solution blending method. The study found that the PVB composite material containing 30 wt% GN achieved a TC of 4.521 W·m^−1^·K^−1^, which is 20.55 times that of pure PVB [170]. In summary, the application of TCPCs in the solar sector not only enhances the efficiency of solar thermal storage systems but also optimizes the thermal management of photovoltaic cells, improving their conversion efficiency and lifespan. As materials science and nanotechnology continue to advance, these new materials are set to play an increasingly significant role in solar technology, driving the utilization and development of renewable energy.

### 4.5. Personal Thermal Management

**Figure 12:**
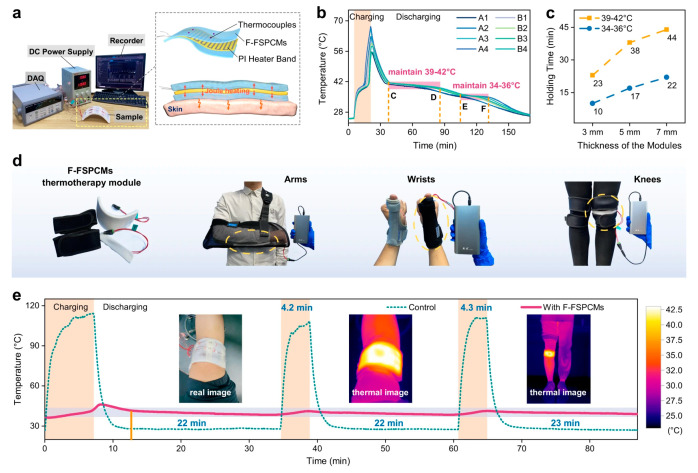
(**a**) Experimental setup for the evaluation of heat storage/release of the F-FSPCMs module. (**b**) Surface temperature of the F-FSPCMs module during a heat storage/discharge cycle (7 mm thickness). (**c**) Temperature holding time of the F-FSPCMs module with the same heat supply for 3 mm, 5 mm and 7 mm thickness. (**d**) Several types of integrated and portable F-FSPCMs modules for personal thermotherapy. (**e**) Temperature evolution of insulating device based on F-FSPCMs module (5 mm thickness) acting on a knee during charging and discharging [177].

The application of TCPCs in personal thermal management is gaining increased attention, as their integration into textiles and garments effectively facilitates heat transfer between the human body and the environment, aiming to enhance and regulate warming and cooling effects. These composites show great potential in this context. The porous structures in textiles and the combination of fibers and air significantly impact thermal conductivity. In cold environments, composites with low TC help retain warmth, whereas in hot settings, materials with high TC aid in dissipating heat, preventing thermal stress. This versatile application of material properties lays the foundation for developing functional garments tailored to various environmental and activity demands [171,172,173]. Soong et al. have developed a multilayered BN-GNP/TPU composite film by mechanically blending GNP and BN fillers into a TPU base film. This composite film exhibits a high TC of 6.86 W·m^−1^·K^−1^ with 20 wt% of BN and GNP, nearly 30 times that of pure TPU. After 5000 bending cycles, its TC remains at 6.25 W·m^−1^·K^−1^, and it maintains 6.85 W·m^−1^·K^−1^ after 10 wash cycles, demonstrating excellent mechanical stability and hydrophobicity, making it highly suitable for smart cooling garments. Additionally, the composite film exhibits good resistance to bending fatigue and washing, maintaining a TC of 6.25 W·m^−1^·K^−1^ after 5000 bending fatigue tests and 6.85 W·m^−1^·K^−1^ after 10 wash cycles. Furthermore, enhanced thermal stability, cooling, and hydrophobicity of the multilayer BN-GNP/TPU composite film were observed in the resulting composite film [174]. Huang et al. proposed a direct strategy for fabricating dual-functional wearable Janus fabrics by electrospinning TPU elastomers onto cellulose cotton fibers while loading thermal conductive BN nanoplates. The resulting fabric exhibited excellent thermal management and humidity control characteristics, with a TC of 0.307 W·m^−1^·K^−1^, reducing the total thermal resistance to 10.62 K cm^2^/W. Indoor and outdoor winter experiments showed that the fabric surface in contact with human skin was 2.9 °C and 3.1 °C warmer than regular fabrics, respectively, demonstrating effective heat dissipation capabilities [175]. Xi et al. developed a simple, scalable method to produce breathable nanofiber membranes with high TC and super-hydrophobic properties, aimed at enhancing the thermal management of personal cooling textiles. Specifically, the in-plane TC reached 17.9 W·m^−1^·K^−1^, the transverse TC was 0.29 W·m^−1^·K^−1^, the water vapor transmission rate stood at 11.6 kg/m^2^·day, the water contact angle was 153°, and the hydrostatic pressure was 32 kPa. These properties make it highly practical and effective for next-generation cooling fabrics, offering a mix of active and passive cooling capabilities [176]. Jing et al. present a chemical cross-linking strategy to develop ultra-flexible polymer-based phase change composites (Figure 12). This method involves a dual 3D crosslinked network of olefin block copolymers (OBCs) and styrene–ethylene–butylene–styrene (SEBS) within paraffin wax (PW). The enhanced C–C bond within the OBC–SEBS networks synergistically improves the mechanical, thermal, and leakage-proof properties of PW@OBC-SEBS. Importantly, the peroxide-initiated chemical cross-linking technique surpasses the limitations of conventional physical blending methods and is applicable across various polymer matrices [177].

In summary, the application of TCPCs in personal thermal management not only enhances thermal comfort but also reduces energy consumption through intelligent design, leading to personalized, energy-efficient thermal management solutions [178,179]. The development of these technologies marks a step toward a more intelligent and efficient future in personal health and comfort management through functional textiles and garments.

### 4.6. Aerospace

The application of TCPCs in aerospace is a testament to the advances in modern technology. These materials play a crucial role in the design and operation of spacecraft and aircraft, particularly in managing heat within extreme temperature conditions. Ensuring efficient thermal management is a significant engineering challenge, crucial for maintaining performance and extending the lifespan of aerospace equipment. In this context, TCPCs, due to their light weight, high strength, and superior thermal conductivity, are increasingly preferred over traditional metal materials [180,181,182]. These composites have been enhanced by the incorporation of inorganic fillers like carbon nanotubes, graphene, and metal oxides, facilitated by advances in nanotechnology. This not only optimizes heat dissipation but also enhances electromagnetic shielding and corrosion resistance, making these materials viable for use in advanced aerospace applications. For instance, they are widely used in manufacturing the external shells, wings, and other critical structural components of spacecraft, enhancing the overall mechanical properties while significantly reducing weight.

In spacecraft thermal management systems, these efficient thermal materials ad-dress the enormous temperature differentials created by one side being exposed to sunlight and the other facing the cold void of space. Adequate TC ensures temperature equilibrium, preventing overheating that could lead to electronic equipment failure. Additionally, advancements in micro- and nanoscale technologies have led to the development of new microfluidic cooling systems and encapsulation structures that further enhance thermal management efficiency. Wang et al. enhanced the in-plane orientation of pitch-based carbon fibers within TPU composite films through solution blending and hot-pressing techniques, successfully creating films with excellent anisotropic TC and high EMI shielding performance. The studies show that with 30 vol% filler, the TPU composite films at 0.2 mm thickness achieved an in-plane TC of 15.11 W·m^−1^·K^−1^ while providing more than 29 dB of EMI shielding. Through subsequent lamination and hot pressing, the in-plane TC of the laminated composite film increased with the number of layers, reaching up to 17.69 W·m^−1^·K^−1^ for a ten-layer laminate. These laminates also demonstrated excellent EMI shielding effectiveness and good mechanical properties across the 8.2–12.5 GHz frequency range [183]. Ryu et al. used lyotropic liquid crystal and wet-spinning processes to develop composite fibers of aromatic polyamide and BN nanotubes (BNNTs). These uniaxially oriented one-dimensional composite fibers are continuous and flexible, enabling excellent thermal stability (up to 479 °C) and significant thermal neutron shielding performance (0.73 mm^−1^). Due to the dense packing and uniaxial orientation of the fibers, their TC reached 7.88 W·m^−1^·K^−1^. These characteristics make this composite textile a promising material for space applications, providing effective radiation and thermal protection for astronauts and electronic equipment [184]. In the future, as nanotechnology and materials science continue to advance, the prospects for TCPCs will broaden further. These materials not only meet the demands of spacecraft for lightweight, high strength, and high TC but will also drive innovation and development in spacecraft thermal management systems [185,186,187,188,189]. In summary, the development of TCPCs has not only enhanced the performance and reliability of spacecraft but has also pushed the entire aerospace field towards higher technological levels, demonstrating the significant potential of modern materials science in applications within extreme environments.

### 4.7. Others

TCPCs are displaying extensive application potential across various emerging fields, notably within crucial technologies such as building materials, transportation, and medical devices, due to their exceptional TC and mechanical properties.

In the realm of construction, TCPCs play a pivotal role as green and smart buildings rise in prominence. These materials are key in maintaining stable indoor temperatures and enhancing energy efficiency. They are utilized in the production of efficient insulative and conductive building materials, which regulate the internal distribution of heat within structures. This not only increases energy utilization efficiency but also reduces the energy consumption of heating, ventilation, and air conditioning systems, aiding in energy conservation and emission reduction efforts [190,191].

In the transportation sector, TCPCs show immense potential. Modern automotive and railway systems require efficient thermal management systems to ensure stability and safety. Many critical components in these systems benefit from the advanced thermal management properties of these composites, helping to keep vital parts functioning within optimal temperature ranges, thus ensuring the safety and efficiency of the transportation infrastructure [192,193,194].

The application of TCPCs in medical devices is also noteworthy. Many medical devices generate significant amounts of heat during operation, necessitating efficient cooling solutions to ensure the devices operate correctly and safely. These composites are used to create effective thermal management components, such as conductive pads and cooling plates, which help maintain a stable operating temperature for medical equipment. This not only enhances the reliability and lifespan of the devices but also ensures patient safety [195,196,197].

As advancements in nanotechnology and materials science continue, TCPCs are set to broaden their impact further across various domains. These materials not only meet modern technology’s demands for high TC and mechanical strength but also enhance the performance and reliability of various devices through intelligent design.

## 5. Conclusions and Outlook

### 5.1. Conclusions

In this comprehensive review, we have thoroughly examined the development of TCPCs, detailing the advancements in this field, elucidating the thermal conduction mechanisms of these materials, and discussing factors that influence their thermal properties, such as the inherent characteristics of the polymers, interface thermal resistance, and thermal performance of fillers. Additionally, methods to enhance the TC of polymer composites have been categorized and summarized. Lastly, this article highlights the applications of these materials in emerging areas such as flexible electronics, personal thermal management, and aerospace.

### 5.2. Outlook

Despite the progress made with TCPCs, several challenges persist. Future developments should be application driven, focusing on the following breakthroughs:

Development and Application of Novel Fillers: With continual advancements in technology, the development of new, highly efficient thermal fillers, such as novel two-dimensional materials and three-dimensional network structure fillers including black phosphorus and MXenes, is expected. These materials are becoming research focal points due to their unique thermal conduction mechanisms and structural properties. Through the further development of functionalization and surface treatment techniques, it is possible to optimize the interface bonding between fillers and the polymer matrix, achieving higher thermal conduction efficiencies, particularly crucial for electronic packaging and energy storage systems.

Multi-scale Composite Design: Future designs of TCPCs will likely emphasize optimization of multi-scale structures. From the nano to the macro level, fine control over the dispersion, orientation, and network structure of fillers can effectively construct thermal conduction pathways. Additionally, structural design and interface engineering of materials will be key to enhancing overall thermal conductivity, necessitating a combination of physical and chemical methods along with the use of computational modeling and simulation technologies to predict and optimize material designs.

Efficient Manufacturing Processes and Scalable Production: As the demand for TCPCs continues to grow, developing efficient and cost-effective manufacturing processes will become a research focus. New technologies, such as 3D printing and electrospinning, are expected to facilitate the efficient fabrication and scalable production of these materials. Moreover, optimizing process parameters during manufacturing and improving production equipment are crucial for enhancing production efficiency and material consistency.

Multifunctionality and Performance Enhancement: Future TCPCs will not only require excellent TC but also need to possess electrical insulation, mechanical strength, and environmental stability (such as thermal stability). This multifunctionality is particularly prominent in the electronics and automotive industries. Through functional design and comprehensive performance optimization, high-performance composite materials that meet specific application needs can be developed. The high loading of fillers not only leads to agglomeration but also negatively impacts the mechanical properties and thermal conductivity of the composites. Although constructing thermal conductive networks through modification, hybridization, or macroscopic construction can enhance thermal conductivity, the filler content in composites still needs to exceed 20 wt% to achieve high thermal conductivity. Generally, the mechanical threshold for polymer composites is within 10 wt%, and the addition of fillers continues to degrade the mechanical properties of the polymer. Therefore, achieving an effective combination of properties at low filler content remains a significant challenge that needs to be addressed.

In-depth Study of Thermal Mechanisms: Although significant progress has been made, the thermal mechanisms of TCPCs still require deeper investigation. In basic theoretical research of composite materials, the forms of thermal conduction are simplistic, and theories of thermal conduction lack depth. Further exploration of thermal mechanisms, multi-scale phonon conduction, and phonon transmission at interfaces is needed. Additionally, the precise mathematical modeling of interface thermal resistance, effective testing methods, and related measurement equipment are lacking. By utilizing advanced characterization techniques and computational simulations, a better understanding of the interactions at the interface between fillers and the polymer matrix and their impact on thermal conduction can be achieved. Further research into the specific impacts of the distribution and orientation of fillers within the polymer matrix on TC will provide theoretical support and experimental basis for designing more efficient TCPCs.

Overall, research on TCPCs is moving towards more efficient, smarter, and more environmentally friendly directions. With the discovery of new materials and application of new technologies, these materials are expected to find broader applications in industries such as energy, electronics, and automotive, providing effective solutions for thermal management challenges.

## Figures and Tables

**Table 1:** Strategies to enhance the TC of polymer composite materials.

Strategies	Polymer Matrix	Fillers	Enhancement in *k* (%)	*k* (W m^−1^ K^−1^)	Ref.
Interfacial Functionalization	PI	Graphene oxide	820	7.13	[99]
	PVA	BNNS	30.6	1.909	[100]
	Natural rubber	CF	71.3	0.218	[101]
	TPU	BN and CNT	210	4.52	[102]
	Epoxy natural rubber	Al_2_O_3_ and BN	370	0.5147	[103]
Microstructural Design	Cyclic butylene terephthalate	Graphene nanoplatelets	1518.8	8.094	[78]
	Cellulose	BNNS	3000	20.41	[87]
	PDMS	C@BNNS	1600	4.09	[89]
	PI	BNNS	4773	13.1	[91]
	Epoxy resin	CF	17100	32.6	[95]
	Cellulose	BNNS/Ti_3_C_2_T_x_ MXene	/	19.97	[96]
	Polyurethane	BN	85.0	39.0	[105]
	Poly(p-phenylene-2,6-benzobisoxazole)	MXene	164	42.2	[107]
